# Chitosan-Based Scaffold for Mineralized Tissues Regeneration

**DOI:** 10.3390/md19100551

**Published:** 2021-09-28

**Authors:** Teerawat Sukpaita, Suwabun Chirachanchai, Atiphan Pimkhaokham, Ruchanee Salingcarnboriboon Ampornaramveth

**Affiliations:** 1 Research Unit on Oral Microbiology and Immunology, Department of Microbiology, Faculty of Dentistry, Chulalongkorn University, Bangkok 10330, Thailand; teerawatsu@nu.ac.th; 2 Center of Excellence on Petrochemical and Materials Technology, Chulalongkorn University, Bangkok 10330, Thailand; suwabun.c@chula.ac.th; 3 Bioresources Advanced Materials (B2A), The Petroleum and Petrochemical College, Chulalongkorn University, Bangkok 10330, Thailand; atiphan.p@chula.ac.th; 4 Department of Oral and Maxillofacial Surgery, Faculty of Dentistry, Chulalongkorn University, Bangkok 10330, Thailand

**Keywords:** chitosan, scaffold, biomaterials, bone tissue engineering, regenerative dentistry

## Abstract

Conventional bone grafting procedures used to treat bone defects have several limitations. An important aspect of bone tissue engineering is developing novel bone substitute biomaterials for bone grafts to repair orthopedic defects. Considerable attention has been given to chitosan, a natural biopolymer primarily extracted from crustacean shells, which offers desirable characteristics, such as being biocompatible, biodegradable, and osteoconductive. This review presents an overview of the chitosan-based biomaterials for bone tissue engineering (BTE). It covers the basic knowledge of chitosan in terms of biomaterials, the traditional and novel strategies of the chitosan scaffold fabrication process, and their advantages and disadvantages. Furthermore, this paper integrates the relevant contributions in giving a brief insight into the recent research development of chitosan-based scaffolds and their limitations in BTE. The last part of the review discusses the next-generation smart chitosan-based scaffold and current applications in regenerative dentistry and future directions in the field of mineralized tissue regeneration.

## 1. Introduction

Tissue engineering by implanting artificial materials has become one of the most highly investigated scientific fields and can be used in regenerative medicine. The primary purposes of tissue engineering can be categorized as restoring, replacing, maintaining, or enhancing the function of different types of biological tissues [1,2]. Chitosan, a natural-based biopolymer, is a deacetylated form of chitin, a major by-product of crustacean shells. Currently, chitosan has attained enormous attention in several industrial applications, including biomedicine, antibacterial food coating, and the pharmaceutical and cosmetic industries [3,4]. Chitosan has received significant interest in bone regeneration [5] because it presents outstanding properties, such as being environmentally friendly, good biocompatibility, sustained drug release, biodegradable, and antimicrobial effects. Chitosan can be used in different biopolymeric composite materials [6]. The purpose of this review is to present an overview of the recent developments, current applications, and future directions of chitosan-based scaffolds in the field of mineralized tissue regeneration.

## 2. Bone Tissue Engineering

Bone is a complex dynamic living tissue that undergoes regrowth and self-repair by modeling and remodeling processes [7]. Bone tissue is responsible for several functions in the human body, e.g., support the body structurally, withstand load bearing, protect the vital organs, and provide an environment for bone marrow [8]. However, the capacity of bone to heal a defect and restore function to an injured bone is often insufficient, especially in a large bony defect. The term tissue engineering, which combines materials science, mechanical engineering, and biology, was first introduced in 1988 [9]. BTE is a research area concerning inventing novel implantable bone substitute materials for critical-sized bone defects that cannot spontaneously heal and aiming to overcome the drawbacks of the current clinical bone disease treatments [1]. The conventional bone tissue engineering model requires three components combining osteogenic stem cells with bioactive molecules (growth factors, genes, and drugs) and seeding them onto three-dimensional (3D) biomaterial scaffolds. These scaffolds are an osteogenic implantable material that can create an ideal environment to accelerate new bone formation and induce new functional tissue integrated into the host bone without causing any adverse reaction. These three components (osteogenic stem cells, bioactive molecules, and scaffolds) are known as the tissue engineering triad.

## 3. BTE Scaffold

**Figure 1:**
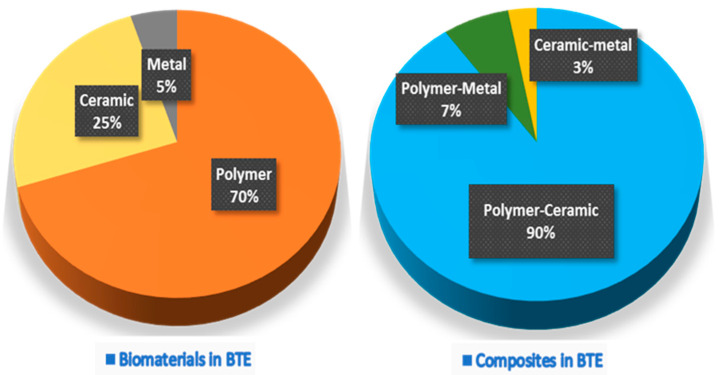
The proportion of pure biomaterials and their composites used in various BTE applications.

An essential aspect of BTE is developing implantable scaffolds that contribute to bone regeneration. Because the cells alone cannot grow in a 3D manner, BTE scaffolds allow the adherence of osteogenic stem cells and provide a suitable environment for the osteogenic cells to differentiate and regenerate new bone. Scaffolds are usually subdivided into three classes based on their original material base, i.e., polymer, ceramic, and metal scaffolds [10,11,12]. The Web of Science^®^ database from 2017 to 2021 represented by 7915 articles shows that polymer scaffolds remain the most commonly developed, followed by ceramics, and are often used in polymer–ceramic combinations as a composite scaffold (Figure 1). Each scaffold type and its combinations have different advantages and disadvantages (Table 1).

**Figure 2:**
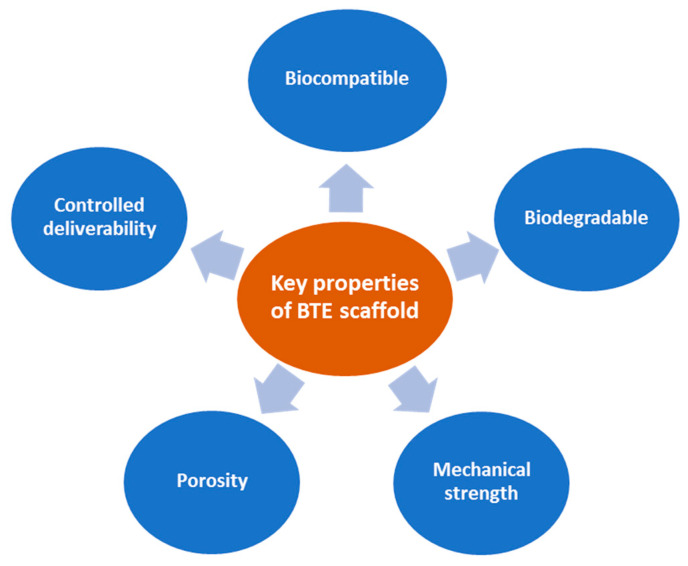
Fundamental properties of ideal BTE scaffolds.

Ideal BTE scaffolds should have specific fundamental properties (Figure 2) to use as a bone-inducing material: (1) Biocompatible, i.e., the material is compatible with living tissue and similar to the native extracellular matrix (ECM). Biocompatible scaffolds do not produce toxic by-products or induce an immune response when exposed to the body. (2) Biodegradable, i.e., the scaffold thoroughly breaks down in a predictable time, concurrent with the regeneration of new bone. (3) Strong mechanical properties to support the applied load transfer during the degradation period. (4) Interconnected porosities with pores ranging from 200 to 350 μm for successful diffusion of essential nutrients, waste transfer, and angiogenesis. (5) Controlled deliverability for releasing the appropriate dose of bioactive molecules (growth factors, genes, or drugs) directly in the desired tissue area [9,10,11,13,14].

## 4. Chitin and Chitosan

**Figure 3:**
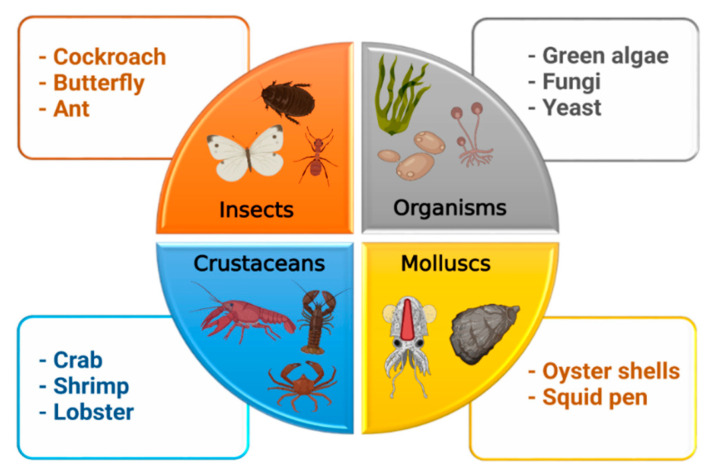
Sources of chitosan.

Chitin (β-(1→4)-poly-N-acetyl-D-glucosamine) is the second most abundant long-chain aminopolysaccharide polymer occurring in nature after cellulose and was first identified in mushrooms in 1811 [4,16]. Although chitin provides strength to the cell wall of some fungi, it is present in the cuticles or exoskeletons of insects, arthropods, mollusks and is mainly isolated from crustaceans, including crab, lobster, crayfish, king crab, and shrimp (Figure 3). For biomedical applications, chitin in the solid-state can be converted through enzymatic or chemical deacetylation to its most well-known fibrous substance derivative, chitosan [3].

**Figure 4:**
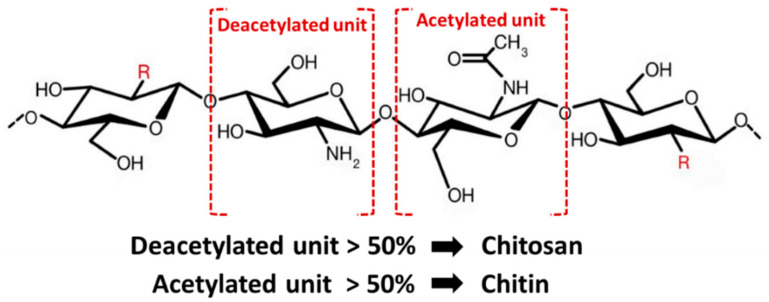
Structure of chitin and chitosan.

Chemically, chitosan is a semi-crystalline de-N-acetylated analog of chitin (its parent polymer), composed of two randomly distributed monomeric units, β-(1→4)-linked D-glucosamine (deacetylated unit, amino unit) and N-acetyl-D-glucosamine (acetylated unit) (Figure 4) [17]. When the β-(1→4)-linked D-glucosamine is the predominant repeating unit, and much higher than 50%, the aminopolysaccharide chain is considered chitosan [18]. The glucosamine to N-acetyl-D-glucosamine molar ratio is referred to as the degree of deacetylation (%DD), which can be determined by NMR spectroscopy, and the %DD in commercially produced chitosan ranges from 50% to 95%. Depending on the chitin source and preparation process, its molecular weight ranges from 300 to more than 1000 kilodaltons [19]. In its crystalline form, very few solid-state chitosans have acceptable solubility in water and most organic solutions above pH 7. In contrast, in acidic solvents, the protonated free amino groups on glucosamine make chitosan soluble [20,21]. Chitosan has three vital functional groups consisting of an amino group (NH2 at C-2), abundant primary hydroxyl groups (OH at C-6), and secondary hydroxyl groups (OH at C-3) [22]. These functional groups can easily generate intermolecular hydrogen bonds without disturbing its polymerization and allow modification of chitosan chain copolymerization crosslinked with other polymeric chains, which can manufacture various types of composite scaffolds and make it an attractive candidate for bone tissue repair and regeneration.

Chitosan has several essential properties, including a low-cost crustacean shells source, ease of scaffold processing, being fast and completely biodegradable, having antibacterial activity, being nonantigenic, displaying high osteoconductivity, displaying high porosity with the appropriate pore size distribution, a controlled drug delivery, and biocompatibility with almost all human tissues. These properties make chitosan attractive for a wide variety of applications, such as BTE scaffolds [3,4,17,20]. Moreover, chitosan has a chemical structure similar to glycosaminoglycans (GAG), the major component of bone′s ECM. Moreover, chitosan has become popular as a BTE scaffold because it can be easily shaped into various shapes, including 3D porous scaffolds, two-dimensional membranes/fibers, nanoparticles, and hydrogels [9,17,23,24]. Thus, chitosan scaffolds can be constructed in the shape of the bone defect.

## 5. Processing of Chitin and Chitosan for BTE

**Figure 5:**
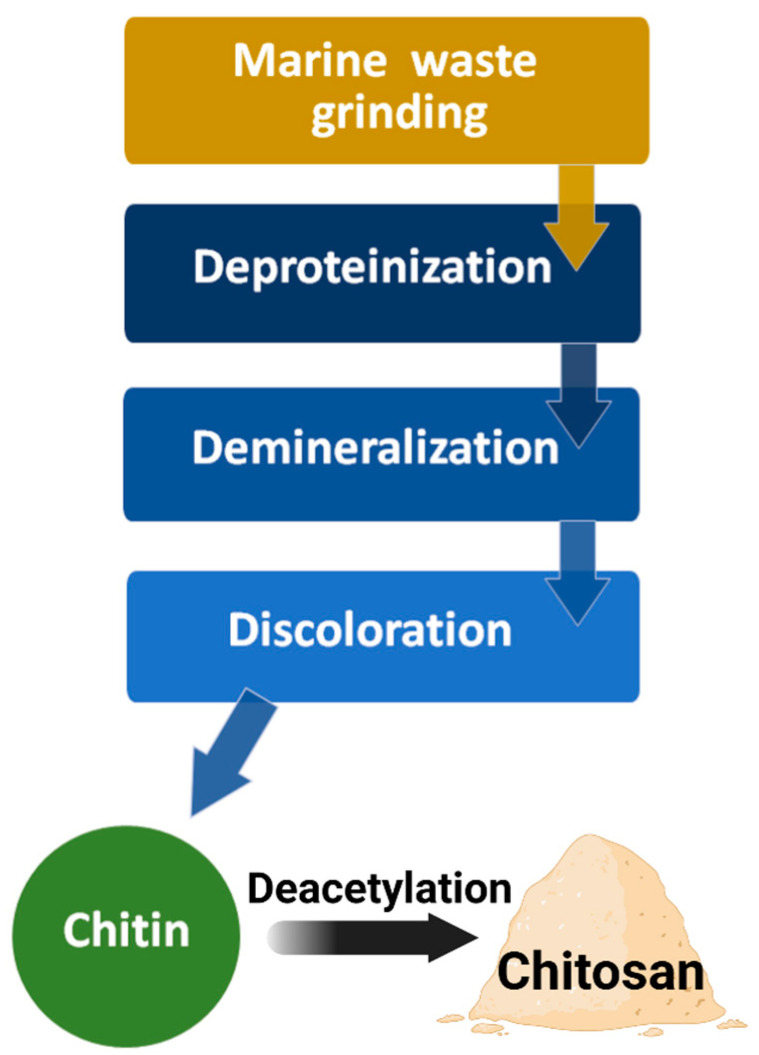
Diagram of chitin and chitosan processing.

The most common raw material for chitin processing is crustacean shells, such as Ectes japonica (red crab), Penaeus monodon (Asian tiger shrimp), and Pandalus borealis (caridean shrimp). The industrial production of chitin from natural resources includes four steps (Figure 5) [3,23]. First, grinding the crustacean shells in a mill; second, deproteinization to remove the protein and oil in an alkaline solution at 100 °C for 4 h; third, demineralization by treating it with hydrochloric acid or sulfuric acid to remove calcium carbonate; finally, treatment with an inorganic solvent (sodium hypochlorite or hydrogen peroxide) for discoloration, washing in hot water, and grinding the particles into the appropriate size to obtain chitin powder.

In the next step of chitosan production, chitin is converted into chitosan via an enzymatic or chemical deacetylation reaction. The most common conventional technique is treating the chitin powder with a high concentration of sodium hydroxide at high temperatures (>80 °C) for 2–6 h. During this chemical deacetylation reaction, most of the acetyl groups on the long-chain polymer are removed and are converted to β-(1→4)-linked D-glucosamine (deacetylated unit, amino unit). Finally, the obtained chitosan is purified by neutralization, washing, and drying.

Several conventional techniques have been used to fabricate chitosan into a porous structure, including freeze-drying, gas foaming, solvent casting/particulate leaching (SCPL), electrospinning, and 3D-printing/rapid prototyping/bioprinting [25,26,27]. Scaffolds made from chitosan can serve as temporary structures for osteogenic cell activities and increase the new bone formation rate. Each fabrication technique has many pros and cons. The ideal fabrication technique has not yet been discovered. Several limitations need to be addressed (Table 2). The requirements of the bone defect dictate the appropriate fabrication technique or whether techniques need to be combined.

## 6. Applications of Chitosan Scaffolds and Their Limitations in BTE

**Figure 6:**
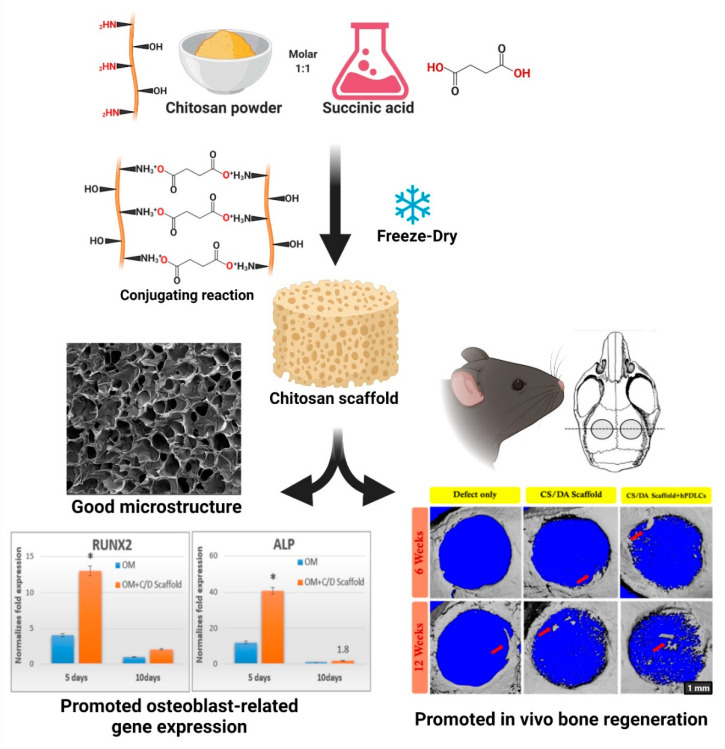
Schematic representation of a novel chitosan scaffold fabrication technique using multifunctional carboxylic acid. This scaffold has the appropriate physicochemical properties and induced new bone formation in a critical-size mouse calvarial defect model. Adapted with permission from reference [32].

Up to date, it has been stated that chitosan has demonstrated very good osteoconductivity, it allows obtaining desirable shapes easily, promotes osteogenic differentiation and mineralization, and prevents inflammatory response or inflammation reaction [28,29,30]. These properties make chitosan an attractive candidate to use as a scaffold sponge for regenerative bone therapy and orthopedic applications. Our team has developed a novel chitosan scaffold fabrication technique using multifunctional carboxylic acid instead of dialdehyde as a crosslinker [31]. Dicarboxylic acids, especially succinic acid, displayed dual functions, protonation for chitosan powder dissolution and crosslinking via amide bond via a conjugating reaction. This method has fewer steps and is an environmental fabrication technique compared with the conventional technique. The in vitro characterization revealed that the chitosan scaffold had appropriate physicochemical properties, mechanical properties, and biocompatibility. Moreover, this novel scaffold can serve as a template for human periodontal ligament cell seeding and enhanced in vivo bone regeneration in a mouse calvarial defect model [32]. These findings strongly suggest that the chitosan scaffold is an appropriate material for use in BTE (Figure 6).

**Figure 7:**
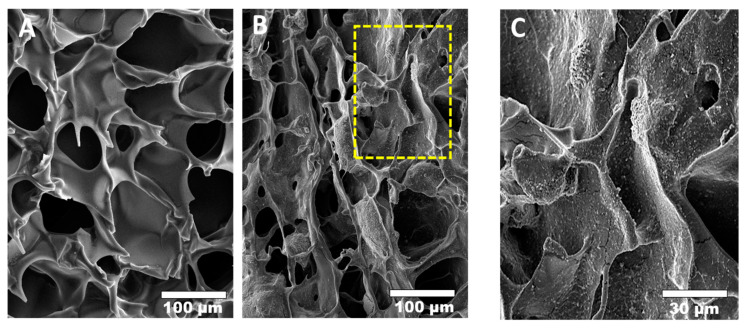
The precipitation of calcium and phosphate ions on the surface of the chitosan scaffold. (**A**) pure chitosan scaffold; (**B**) chitosan/BCP scaffold; (**C**) high magnification of B.

Although a pure chitosan scaffold has many advantages, its mechanical strength and degradation rate are inadequate, especially in acidic environments or in the human body where lysozymes are present [33,34,35]. In scaffold fabrication, these problems have been solved by combining or incorporating the chitosan scaffolds with other functional bioceramics or polymers, such as HA, β-TCP, BCP, hyaluronic acid, and collagen, which increase the hardness of chitosan by distributing the applied stress [36], resulting in improved mechanical and biological properties and making them suitable for BTE. Chitosan/bioceramic composites have become one of the most popular combinations because they rapidly precipitate mineral ions, such as calcium and phosphate ions on the surface of chitosan scaffolds, establishing a robust mechanical interface (Figure 7). A chitosan/HA (60% and 70% *v*/*v*) scaffold has been developed using freeze-drying. These composite scaffolds supported human mesenchymal stem cell (hMSC) proliferation and differentiation.

Moreover, this scaffold reduced the production of anti-inflammatory cytokines [37]. Chatzipetros et al. reported the histological and histomorphometric effects of a nano-hydroxyapatite/chitosan (25% *w*/*w*) scaffold in an animal model, demonstrating increased new bone formation and the total number of osteocytes in rat calvarial defects [38]. Zhang et al. also developed mineralized collagen and chitosan electrospun nanofibers loaded with berberine. These hybrid polymer fibers had the appropriate mechanical properties, induced the attachment and proliferation of an osteoblast cell line, sustained release of a bioactive drug, and increased new bone formation in a rat femoral bone defect model [39]. Alginate is another candidate biomaterial for BTE; however, its major disadvantage is poor cell adhesion and migration. A study reported using chitosan/alginate coatings on electrospun fibers as pH responsiveness for the sustained release of ibuprofen. The results indicated that the presence of polycationic chitosan regulated the release of ibuprofen [40]. In recent decades, 3D-printing technology has expanded to bone regenerative medicine to construct 3D bone substitute materials with controllable geometry. Aydogdu et al. designed 3D-bioprinted PLA/β-TCP/chitosan loaded with amoxicillin. This printed scaffold exhibited favorable mechanical characteristics and no cytotoxicity to human osteosarcoma cells [41]. Overall, combining a chitosan scaffold with other functional bioceramics or polymers is a promising strategy to develop excellent biomaterials for bone repair and regeneration.

## 7. Use of Chitosan Scaffolds in Growth Factors/Genes/Drug Delivery

Drug delivery is a broad field of research on the novel materials, storage systems, and technologies that enable the introduction of a pharmaceutical compound into patients to achieve its appropriate therapeutic effect by altering the drug′s bioavailability in a controlled manner of rate, time, and place of drug release [42,43]. A major drawback of chitosan is its low osteoinductivity compared with bioceramics or commercial calcium-phosphate-based bones substitutes. The easiest way to solve this problem is by adding osteoinductive molecules into the chitosan structure. 

Due to their hydrophilic nature, chitosan-based polymers can be used as a starting material for incorporating bioactive molecules into drug delivery systems [44,45]. For bone regeneration, the most popular osteogenic molecules with promising clinical outcomes used for drug delivery systems include bone morphogenetic protein 2 (BMP-2), bone morphogenetic protein 7 (BMP-7), transforming growth factor-beta 1 (TGF-β1), and vascular endothelial growth factor (VEGF) [46,47,48,49]. Wang and coworkers developed a diatomite/chitosan composite scaffold loaded with BMP-2, in which each component had individual osteogenic activity and exhibited a slow release of BMP-2 [50]. In contrast, instead of being used as a base material, chitosan can be prepared as nanoparticles for drug incorporation, and these can be loaded into another biomaterial scaffold. Liu et al. developed silk fibroin scaffolds incorporated with chitosan nanoparticles that delivered TGF-β1 and BMP-2. This combination demonstrated remarkable biocompatibility. Moreover, bioactive molecules from chitosan nanoparticles can be continuously release up to 7 days [51].

The slightly polycationic charged chitosan structure allows it to interact with DNA or siRNA and can function as a nonviral vector. This property makes chitosan an attractive candidate to use as gene-activated scaffolds or matrices (GAM) for gene delivery strategies [52,53,54]. Lu et al. reported the development of a pure chitosan scaffold embedded with plasmid-DNA nanoparticles encoding TGF-β1. The porous chitosan scaffold acted as a 3D carrier for the nanoparticles. Interestingly, this novel GAM scaffold demonstrated a sustained release of nanoparticles up to 120 days and increased chondrocyte TGF-β1 expression and proliferation [55]. To understand better about the development of chitosan-based scaffolds and their properties, we listed the recent reports of chitosan along with bioactive drugs/cells employed in BTE (Table 3).

## 8. Next-Generation Chitosan Scaffold for BTE

**Figure 8:**
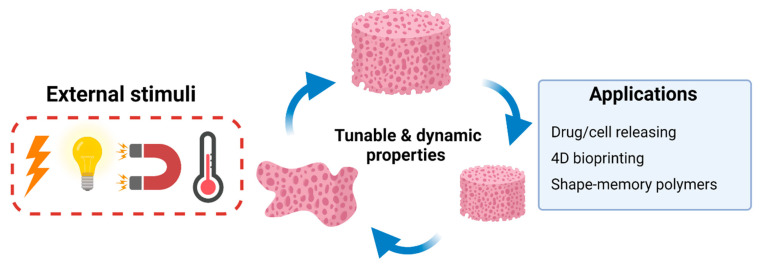
Schematic representation of the stimuli-responsive chitosan scaffolds and their applications in BTE.

A new generation of BTE scaffolds seeks to further improve bone regeneration ability through the development of a “smart” scaffold, which is defined as a new class of scaffold that actively respond to external or internal stimuli such as mechanical forces, magnetic forces, temperature, pH levels, electrical fields, and enzymes, leading to a change in their shape, volume, or physical structure (Figure 8). Smart scaffolds can display adaptable and dynamic properties, allowing the researcher to manipulate scaffold properties in the desired direction [56,57]. These new generation of chitosan scaffolds are primarily employed in the field of BTE as an active appliance for precision drug or stem cell delivery and shape memory polymers (SMP) [57]. For example, Nafee et al. reported the development of chitosan-based hydrogels, which deliver bone resorption inhibitor alendronate. The hydrogel showed thermoresponsive ability and controlled drug release over 45–65 days with less inflammation and faster maturation of granulation tissue [58]. Shape-memory polymer is one of the stimuli-responsive polymers which can change their shape, size, and mechanical properties with the activation of external stimuli [59]. Hu et al. developed poly (lactic acid-co-trimethylene carbonate)/chitosan composite scaffold which have thermo-responsive property. They showed it rapidly recovered its original shape when stimulated with body temperature (within 5 minutes). Moreover, the composite scaffolds exhibited excellent biocompatibility and enhanced adhesion of MC3T3-E1 cells [60]. Fu et al. developed a chitosan/polyurethane cryogel scaffold that possesses switchable shape-memory property. In 4 °C water, this scaffold recovered its original shape, while in 37 °C water, it transformed to a long strip shape [61]. In summary, the shape-memory chitosan scaffold represents a potential use for BTE and minimally invasive bone surgery. Combining 3D printing technology with smart scaffolds has created a promising research field known as four-dimensional (4D) bioprinting. The 4D bioprinting scaffold possesses the dynamic ability to change its shapes under stimuli and adapt to the native microenvironments of the bone defect [62]. For example, Seo and coworkers used 4D printing technology to create a hydroxybutyl methacrylated chitosan scaffold. The results showed that the scaffolds have the thermoresponsive ability and tunable physicochemical properties according to temperature [63]. Indeed, new generation smart chitosan-based scaffolds display excellent ability for improved outcomes of bone regeneration.

## 9. Use of Chitosan Scaffolds in Regenerative Dentistry

The periodontium comprises specialized tissues that surround, attach, and support the teeth. It consists of four components: gingiva, periodontal ligament, cementum, and alveolar bone. Severe inflammation of the periodontium is associated with progressively worsening periodontal bone defects resulting in tooth mobility or loss [64,65]. Several biomaterials have been developed as a 3D scaffold to provide an appropriate microenvironment and facilitate good periodontal regenerative outcomes [65,66]. Chitosan-based scaffolds are considered a promising biomaterial for periodontal regeneration because of their antimicrobial, biocompatible, and osteoconductive effects [67]. In addition, our reports demonstrated that chitosan-based scaffolds significantly promoted periodontal ligament cell attachment and in vitro osteoblast-related gene expression [31,32].

**Figure 9:**
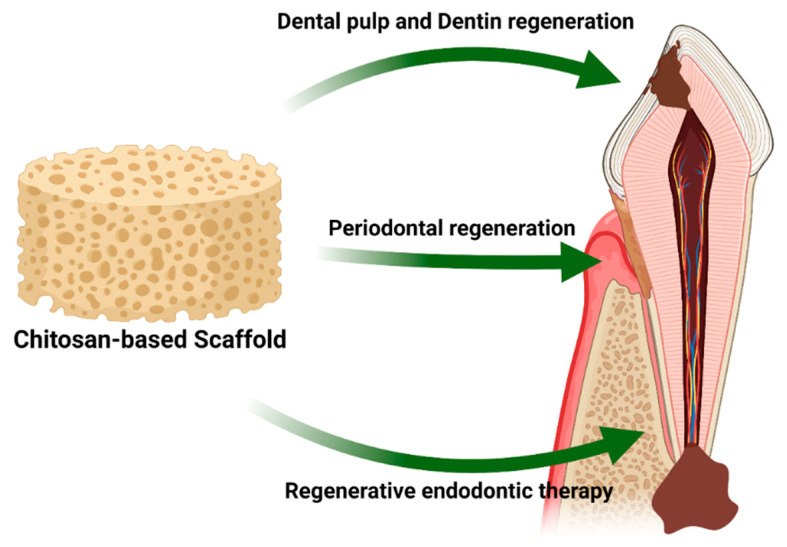
Schematic representation of applications of chitosan-based scaffolds in regenerative dentistry.

Moreover, Liao and coworkers developed a mesoporous hydroxyapatites/chitosan scaffold and evaluated its biological properties. They found that the composite scaffold inhibited the growth of Fusobacterium nucleatum and Porphyromonas gingivalis, and promoted new bone and cementum formation [68]. Chitosan-based scaffolds have been widely used in regenerative dentistry for alveolar bone regeneration, dentin regeneration, and regenerative endodontic therapy (Figure 9). A previous study confirmed that chitosan-based scaffolds provided 3D support for tertiary dentinogenesis in a dog model [69]. Soares et al. developed a calcium-linked chitosan scaffold. It was shown that this combination demonstrated sustained calcium ion release for 21 days and promoted dental pulp cell attachment and odontoblastic-related gene expression [70]. Currently, treatment of pulp necrosis in immature permanent teeth has evolved from conventional root canal treatment towards regenerative endodontic therapies (RET) to regenerate the dental pulp and continue root development [71]. An antibiotic-loaded chitosan-based scaffold was investigated by Aksel et al. for in vitro RET, and the results demonstrated that the scaffold exhibited an antibacterial effect and induced dental pulp stem cell alkaline phosphatase activity [72].

## 10. Conclusions and Future Trends

In the context of BTE, several studies highlighted the potential use of chitosan as a 3D scaffold for BTE because of its positive charge, biocompatibility, osteoconductivity, and biodegradability. The present review provides an updated summary of the basic knowledge, fabrication techniques, and clinical uses of chitosan-based scaffolds for BTE. This review has demonstrated that chitosan′s properties make it a promising biomaterial for mineralized tissue regeneration. However, despite their advantages, pure chitosan scaffolds have shown poor mechanical properties, rapid degradation rates, and low osteoinductivity, which limit their use. Therefore, to solve these problems, researchers should focus on combining chitosan with other biomaterials or bioactive molecules to increase their regenerative potential. Moreover, clinical trials are needed to explore the efficacy and safety of chitosan-based biomaterials, which could be an excellent addition to the field of mineralized tissue regeneration.

## Figures and Tables

**Table 1:** Type of biomaterials for BTE scaffolds and their advantages and disadvantages [6,9,12,15].

Material Type	Advantage	Disadvantage	Example Materials
Metal	Biocompatibility Bioinert Good mechanical properties Fatigue resistance	Bioactive molecules cannot be integrated Not biodegradable Metal ion release Low elasticity	Titanium alloy Magnesium alloy Iron alloy
Ceramic	Biocompatibility Osteoinductive properties Good mechanical properties	Low fracture toughness High brittleness Difficult to manufacture Slow degradation	Hydroxyapatite (HA) Calcium carbonate (CC) Dicalcium phosphate (DCP) Octacalcium phosphate (OCP) β-Tricalcium phosphate (β-TCP) Biphasic calcium phosphate (BCP)
Polymer	Biocompatibility Low antigenicity response Easy formability Enzymatic biodegradability Easy chemical modification Crosslinking capacity	Low osteoinductive capacity Poor mechanical properties	***Synthetic polymers*** Polylactic-co-glycolic acid (PLGA) Polylactic acid (PLA) Polyglycolides (PGA) Polycaprolactone (PCL) ***Natural polymers*** Collagen Cellulose Hyaluronan Fibrin Chitosan
Composite	Combines the advantages of each material type	Difficult to fabricate	β-TCP-Chitosan HA-Chitosan HA-Collagen HA-PLGA

**Table 2:** Techniques of chitosan scaffold fabrication for BTE.

Techniques	Description	Advantages	Disadvantages
Freeze-drying	Chitosan solutions are cooled down to a frozen state, allowed to form ice crystals followed by dehydration	Good pore interconnectivity Without high temperatures Few simple steps Easy control of porosity	Small pore size Low porosity Long fabrication time Expensive technique
Gas foaming	Chitosan is placed under pressure with an inert gas, usually carbon dioxide (CO_2_), resulting in the nucleation of gas bubbles within the structure	Organic solvents not required Inexpensive technique	Insufficient pore interconnectivity Insufficient mechanical strength Nonporous external surface
Solvent casting/particulate leaching (SCPL)	Chitosan solution is mixed with water-soluble salt particles and solidified; salt particles are then leached out	Controls the final pore size and porosity Minimal amount of material required Inexpensive technique	Insufficient pore interconnectivity Insufficient mechanical strength -Remaining toxic porogen
Electrospinning	Electrostatic forces are applied to draw charged threads of chitosan solutions into fine chitosan nanofibers	Very fine fiber thickness High surface-to-volume ratio Mimics the ECM structure	Limited cell seeding Mechanical strength and porosity decrease with fiber thickness
3D-printing/ Rapid prototyping/Bioprinting	Computer-aided design (CAD) creates a layer-by-layer 3D chitosan scaffold	Complex 3D construct with controlled architecture and porosity Reproducible Easy incorporation of bioactive molecules	Use of high temperatures Insufficient mechanical strength Low-throughput technology Long fabrication time

**Table 3:** Chitosan-based scaffold in BTE.

Combination of Biomaterials	Bioactive Drugs/Cells	Fabrication Technique	Observations	Ref.
Pure chitosan	hPDLCs	Freeze-dry	In vitro and in vivo experiment No cytotoxicity with hPDLCs Enhanced bone regeneration in mouse calvarial defect model Low mechanical strength Speedy degradation rate	[31,32]
Chitosan/HA	-	Freeze-dry	In vitro experiment Chitosan/HA (60% and 70% *v*/*v*) scaffold can enhance differentiation of hMSC Can modulate the production of proinflammatory and anti-inflammatory cytokines	[37]
Chitosan/HA	-	Freeze-dry	In vivo experiment Chitosan/HA (25% *w*/*w*) scaffold provide suitable osteoconductive property Enhanced bone regeneration in rat calvarial defect model Good biodegradability	[38]
Chitosan/Mineralized collagen	Berberine	Electrospinning	In vitro and in vivo experiment Favorable mechanical properties Enhanced MC3T3-E1 cells proliferation and attachment Enhanced bone regeneration in rat femoral bone defect model Subsequent sustained release of bioactive drug	[39]
Chitosan/Alginate/PLGA	Ibuprofen	Electrospinning	In vitro experiment pH responsiveness for sustained drug release	[40]
Chitosan/PLA/β-TCP	Amoxicillin	3D-bioprinted	In vitro experiment Favorable mechanical properties No cytotoxicity to Saos-2 (human osteosarcoma) cells Increase antimicrobial activity by amoxicillin	[41]
Chitosan/Diatomite	BMP-2	Freeze-dry	In vitro experiment Enhance proliferation and osteogenic differentiation of the mesenchymal stem cells - Slow-release performance of BMP-2	[50]
Silk scaffold /Chitosan nanoparticles	TGF-β1, BMP-2	Freeze-dry	- In vitro and in vivo experiment - Favorable mechanical properties - No cytotoxicity with bone marrow stromal cells - Bioactive drugs from chitosan nanoparticles can continuously release up to 7 days - Enhanced chondrogenesis in a rabbit knee joint cartilage defect model	[51]
Pure Chitosan	Plasmid-DNA Encoding TGF-β1	Freeze-dry	- In vitro experiment - Increased chondrocyte TGF-β1 expression and proliferation - Sustained release of nanoparticles up to 120 days	[55]
